# The Role of Metabolism in the Development of Personalized Therapies in Acute Myeloid Leukemia

**DOI:** 10.3389/fonc.2021.665291

**Published:** 2021-05-19

**Authors:** Vilma Dembitz, Paolo Gallipoli

**Affiliations:** Centre for Haemato-Oncology, Barts Cancer Institute, Queen Mary University of London, London, United Kingdom

**Keywords:** acute myeloid leukemia, metabolism, personalized therapy, leukemic stem cell, drug resistance

## Abstract

Despite significant recent advances in our understanding of the biology and genetics of acute myeloid leukemia (AML), current AML therapies are mostly based on a backbone of standard chemotherapy which has remained mostly unchanged for over 20 years. Several novel therapies, mostly targeting neomorphic/activating recurrent mutations found in AML patients, have only recently been approved following encouraging results, thus providing the first evidence of a more precise and personalized approach to AML therapy. Rewired metabolism has been described as a hallmark of cancer and substantial evidence of its role in AML establishment and maintenance has been recently accrued in preclinical models. Interestingly, unique metabolic changes are generated by specific AML recurrent mutations or in response to diverse AML therapies, thus creating actionable metabolic vulnerabilities in specific patient groups. In this review we will discuss the current evidence supporting a role for rewired metabolism in AML pathogenesis and how these metabolic changes can be leveraged to develop novel personalized therapies.

## Introduction

Acute myeloid leukemia (AML), the most prevalent acute leukemia in adults (1), is a highly heterogeneous disease. Clinically, patients present with symptoms due to blood cytopenias secondary to the bone marrow failure due to the marrow expansion of immature myeloid progenitors with a concurrent block in normal maturation. The accumulation of blasts often causes high peripheral white cell counts and can also lead more rarely to the seeding of leukemic cells in extramedullary tissues (2). AML is a disease of the elderly, with a median age at presentation of over 65 (3). Remission induction treatment in AML consists of standard chemotherapy with a combination of cytarabine and daunorubicin generally followed by further consolidation chemotherapy and possibly a hematopoietic stem cell transplant according to patients risk profile. Such intensive treatment is often precluded to elderly/unfit patients because of its inherent toxicity. Moreover, despite high remission rates with current therapies, the majority of patients will suffer disease relapse which means that only 35 to 40% of adult patients younger than 60 and 5 to 15% of patients who are older than 60 years of age are cured. The outcome in older patients who are unable to receive intensive chemotherapy remains particularly poor, with a median survival of only 5 to 10 months (2).

AML arises either in a hematopoietic stem cell (HSC) or a more differentiated progenitor which has acquired the ability to self-renew indefinitely as a result of specific mutations (4). This cell of origin named leukemic stem cell (LSC) is able to recapitulate disease and generate relapse due to its resistance to standard chemotherapies. AML LSCs can reside in different phenotypically characterized populations in each patient and are able to evolve clonally through the acquisition of subsequent mutations (5). Recent sequencing efforts to identify novel gene mutations have led to further refinement of AML categorization in different subtypes based on their mutation profile and its putative effect on AML pathogenesis. Among the subgroups identified are those carrying mutations in transcription factors/epigenetic regulators, those carrying mutations in genes encoding for components of the spliceosome machinery and cohesin complexes, and cases carrying mutations in signaling genes (6, 7). In parallel with these sequencing efforts, further research in AML biology has highlighted altered transcription/epigenetic dysregulation and abnormal signaling as common themes in many AML subtypes (8). Thanks to the above efforts, we have recently witnessed the development of multiple novel therapies in AML with already nine being FDA approved for use since 2017 (see **Table 1**). Many of these novel therapies, although often not curative, have led to improved outcomes and increased our ability to treat less fit patients more effectively (23). This is because they either specifically target recurrent mutations found in some AML patients or are more effective in specific subgroups thus making them more tailored and less toxic and a first step towards a more personalized therapeutic approach. However, currently only a minority of patients can access true personalized precision therapy based on their genomic profile. Moreover, many of the recurrent mutations found in AML are not ideal therapeutic targets, either because it is not possible to design small molecules interfering with their function (i.e. transcription factors), or because they are loss-of-function mutations whose wild-type function is difficult to restore with a small molecule. As a result, it is important to identify other targetable biological features associated with unique AML subtypes which will allow the development of specific, highly effective and non-toxic therapy for most if not all AML patients. Rewired metabolism has emerged as a new hallmark of cancer (24) and recently there has been increasing evidence supporting its role in AML initiation and maintenance (25). Perhaps more importantly, unique metabolic rewirings have been shown to develop in specific AML subtypes (26), in response to therapy (27) or in specific AML cell populations including LSC (28). Metabolic reactions are carried out by specific enzymes which are targetable with small molecules and many metabolic inhibitors are already in clinical use or clinical development (29). Targeting rewired metabolism might therefore become a precision medicine therapeutic approach based on our knowledge of the specific metabolic vulnerabilities associated with AML subtypes, cell population and/or therapy combinations. Here we will discuss how targeting metabolism might help in fulfilling the promise of personalized medicine in AML.

**Table 1:** Agents recently approved for treatment of AML (9, 10).

Approval (FDA)	Name	Mechanism of action	AML patient group	Possible interactions with metabolic pathways
2020	Oral azacitidine (11)	Hypomethylating agent	Maintenance therapy for adults in first remission	Multiple: Reports of suppression of TCA cycle and OxPhos in combination with venetoclax; as single agent downregulates several metabolic patways in AML cell lines (12)
2018/2019	Ivosidenib (13)⁠	IDH1 inhibitor	Relapsed/refractory or Age ≥75 years or comorbidities precluding intensive therapy	Inhibitor of cytosolic isocitrate dehydrogenase 1
2018	Glasdegib [in combination with low dose AraC (14)]	Hedgehog pathway inhibitor	Age ≥75 years or comorbidities precluding intensive therapy	No major known interactions
2018	Venetoclax [in combination with hypomethylating agent (15)⁠ or low dose AraC (16)]	Bcl-2 inhibitor	Age ≥75 years or comorbidities precluding intensive therapy	Reports of suppression of TCA cycle and OxPhos
2018	Gilteritinib (17)⁠	Multiple TK inhibitor including FLT3	Relapsed/refractory	Reports of synergistic activity of this class of compounds with inhibition of glycolysis, glutaminolysis and ROS scavenging
2017	Midostaurin [in combination with standard chemotherapy (18)]	Multiple TK inhibitor including FLT3	Newly diagnosed	Reports of synergistic activity of this class of compounds with inhibition of glycolysis, glutaminolysis and ROS scavenging
2017	Enasidenib (19)⁠	IDH2 inhibitor	Relapsed/refractory	Inhibitor of cytosolic isocitrate dehydrogenase 2
2017	Gemtuzumab ozogamicin [monotherapy (20)⁠ or in combination with standard chemotherapy (21)]	Anti-CD33 monoclonal antibody (gemtuzumab) conjugated to cytotoxic agent (ozogamicin)	Relapsed/refractory, Newly diagnosed	No major known interactions
2017	CPX-351 (22)⁠	Liposomal formulation of daunorubicin and cytarabine (1:5 ratio)	t-AML or AML with MRC (no age restriction)	No major known interactions

## Metabolic Changes in Patients With Acute Myeloid Leukemia

The association between changes in metabolic status and prognosis or severity of disease in patients with AML has been long known. In hospitalized patients, hyperglycemia is associated with adverse prognosis independently of their cytogenetics profile or advanced disease status, and this effect can be only partially explained by higher risk of sepsis (30). Additionally, higher glucose levels and increased glycemic variability result in lower remission rates and increased mortality in older patients with AML (31). Modified glucose metabolism, presenting as enhanced glycolysis and truncated TCA cycle monitored in serum of cytogenetically normal patients by a panel of six metabolites, could predict inferior prognosis independently of other molecular markers (32). This evidence of a global metabolic shift is in accordance with observations that AML cells modify the systemic regulation of glucose metabolism by inducing insulin resistance and hyperglycemic phenotype to allow for an increased availability of glucose necessary for their own growth (33). However, despite being a good indicator of the relevance of metabolic changes in AML biology, the use of such a generalized approach as the measurement of serum metabolites does not allow for development of any targeted therapeutic strategies, apart from supportive measures like controlling glycemia.

## Metabolic Plasticity of Acute Myeloid Leukemia Cells

It’s been almost a century since Otto Warburg first described aerobic glycolysis as one of the hallmarks of the metabolism of cancer cells (34). AML is not different from other malignancies in its dependency on glycolysis and increased glucose uptake (32, 35, 36). In BCR-ABL and MLL-AF9 leukemia models, already a modest inhibition of glycolysis performed by the deletion of M2 pyruvate kinase isoform (PKM2) resulted in inhibition of leukemogenesis, while sparing the physiological hematopoiesis (37). Inhibition of glycolysis, either through pharmacological inhibition by the use of 2-deoxyglucose (2-DG) or downregulation of glucose transporter 1, increased the sensitivity of AML cells to classically used chemotherapeutics like cytarabine (32, 38, 39). Additionally, 2-DG alone demonstrated strong antileukemic effects in both AML cell lines and primary samples harboring FLT3-ITD and KIT mutations also through inhibition of N-glycosylation and surface expression of the mutant receptor tyrosine kinases (40). Further confirmation for the relevance of glycolysis in AML cell viability comes from a report which showed that in conditions of glucose deprivation, AML cells switch to fructose utilization to fuel the glycolytic pathway by upregulation of fructose transporter GLUT5 which leads to more malignant phenotype and increased chemoresistance (41). Another possible strategy in targeting glycolysis could be through modulation of lactate metabolism, as a recent report has shown that inhibition of lactate transporters MCT1 and MCT4 induced cell death and increased sensitivity to chemotherapy (42).

However, typical descriptions of the Warburg effect, as well as the use of 18-fluorodeoxyglucose as a marker of cancerous cells in positron emission tomography, often lead to a common misconception of cancer cells switching their energy metabolism to glycolysis on the expense of tricarboxylic acid (TCA) cycle and oxidative phosphorylation (OxPhos), while in reality, both pathways are dysregulated and often increased in comparison to healthy cells of origin, which makes them both interesting for potential therapeutic targeting (34). Infact, in AML LSC a specific dependency on OxPhos has been demonstrated. This can be specifically targeted using venetoclax, a Bcl-2 inhibitor which suppresses both TCA cycle and OxPhos, most likely by inhibiting amino acid metabolism which fuels TCA cycle in AML stem cells (28, 43, 44). This is in stark contrast to metabolic properties of healthy hematopoietic stem cells and bulk AML cells that show a far higher glycolytic reserve rendering them less sensitive to agents that inhibit mitochondrial activity (28). Metabolic plasticity of AML LSC nevertheless allows for an escape from metabolic pressure of venetoclax treatment by an increase in fatty acid oxidation (FAO) that continues to drive enhanced OxPhos when amino acid metabolism is inhibited leading to resistance to venetoclax/azacitidine treatment protocol (44, 45), confirming previous experimental results that simultaneous pharmacological inhibition of FAO enhances sensitivity of AML cells to Bcl-2 inihibiton (46). Further support for the dependency of AML LSC on OxPhos has been lent by a recent report showing that chemoresistant AML LSC are better defined by their metabolic characteristics than their immunophenotype, with cytarabine resistant cells being highly dependent on OxPhos and FAO (47). The strong reliance on FAO in AML cells, even independently of exposure to treatment, can be at least partially explained by downregulation of prolyl hydroxylase domain 3 protein (PHD3), an upstream regulator of acetyl-coA carboxylase 2 (ACC2). In physiological conditions, PHD3 and ACC2 activation suppresses FAO in the presence of other nutrients and therefore a prominent downregulation of PHD3 observed in AML patients could lead to this described dependence of AML cells on FAO (48).

Glutamine is a highly abundant non-essential amino acid that can serve as a source of precursors for TCA cycle through conversion to glutamate and α-ketoglutarate (αKG). Glutamine metabolism thus carries a great potential for therapeutic targeting in AML. Indeed, inhibition of glutamine utilization, either through uptake inhibition (49, 50) or a blockade in glutaminolysis (51), resulted in marked anti-leukemic effects in several AML models. Cellular roles of glutamine are, though, much broader then just serving as a metabolic precursor of αKG. Glutaminolysis supplies cells with precursors for synthesis of glutathione, one of the most abundant cellular regulators of redox status, and *de novo* nucleotide synthesis. When used in combination with drugs that increase ROS formation, like arsenic trioxide or homoharringtonine, inhibitors of glutaminase (GLS), the first enzyme in glutaminolysis, significantly decreased the viability of AML cells and leukemia burden in treated mice (52). Glutamine role in *de novo* nucleotide synthesis has been less investigated, although many of classical chemotherapeutics are nucleoside analogues, and there is evidence that resistance to demethylation agents decitabine and 5-azacytidine, both nucleoside analogue prodrugs, could be circumvented by pharmacological inhibition of *de novo* pyrimidine synthesis (53). Targeting of *de novo* purine synthesis can also be achieved through inhibition of one carbon folate pathway (54) or pyrimidine synthesis through inhibition of dihydroorotate dehydrogenase (DHODH) (55–57), which induces differentiation of AML cells. Several clinical trials are currently assessing the efficacy of DHODH inhibitors as potential therapies in AML (clinicaltrials.gov: NCT03760666, NCT04609826, NCT03761069, NCT03404726).

The greatest success in metabolic targeting, and a proof of concept that specific modulation of metabolic pathways could result in therapeutic benefit for specific subgroups of AML patients, is differentiative therapy of patients carrying isocitrate dehydrogenase (IDH) mutations with IDH inhibitors. IDH1 and IDH2 mutations are present in 15–20% of patients with AML (58). The product of the neoezymatic activity of the mutated protein, 2-hydroxyglutarate (2-HG), competitively inhibits αKG-dependent enzymes, such as epigenetic regulator TET2, and induces a hypermethylated phenotype in AML cells that results in differentiation blockade (59). Small molecule inhibitors of both IDH2 (60) and IDH1 (61, 62) reduce the levels of 2-HG and induce differentiation of AML cells, and were able to produce durable remissions in patients when used as single agents (13, 19, 63). IDH2 inhibitor enasidenib and IDH1 inhibitor ivosidenib hence became first FDA-approved metabolism targeting drugs for AML.

Based on this, it is clear that the number of potential metabolic targets in AML is large. The question that naturally arises is how this extensive knowledge of metabolic changes in AML cells can help us identify the subgroups of patients who could benefit the most from modulation of specific metabolic pathways, thus leading to the development of tailored metabolic therapeutic approaches.

## Personalized Approaches to Metabolic Targeting of AML

A recent metabolomics study on bone marrow samples of patients with *de novo* AML showed that when stratified by morphologic properties, genetic markers, differentiation status and European LeukemiaNet (ELN) 2017 risk groups, AML patients demonstrated a strong diversification of their metabolic properties (64). This underlines the potential of molecular markers already in use to serve as a guide to define patient subgroups with specific metabolic vulnerabilities.

Beside the previously mentioned success of targeting dysregulated metabolism of αKG in patients carrying IDH1 and IDH2 mutations, potential metabolic targets have been identified in AML subgroups with other molecular signatures. One of the main regulators of cellular αKG pools, and hence the activity of TET-family of DNA demethylases, is branched-chain amino acid transaminase 1 (BCAT1), the enzyme involved in branched-chain amino acids (leucine, isoleucine and valine) catabolism by transferring α-amino groups to αKG, producing glutamate and corresponding α-ketoacids. In IDH^WT^TET^WT^ patients overexpression of BCAT1 phenocopies the effects of IDH mutation and presents with a decrease in αKG levels and subsequent DNA hypermethylation. Patients with high levels of *BCAT1* showed enrichment for leukemia stem cell signatures and relapsed patients present with an increase in *BCAT1* levels (65). Interestingly, BCAT1 is also involved in leukemic transformation of EZH2-mutated myeloproliferative neoplasms (66), as well as blastic transformation of chronic myeloid leukemia (67), which further emphasizes the potential of this enzymatic pathway for the development of targeted therapies in specific AML subtypes.

The research in amino acid metabolism in AML is currently mostly focused on glutamine because it is a known vulnerability of AML cells, and specific inhibitors of GLS are reported to be safe and well tolerated in a Phase Ib/II clinical trial in patients with advanced myelodysplastic syndrome (68). AML cells carrying the poor prognosis internal tandem duplication (ITD) activating mutation in the FLT3 tyrosine kinase are specifically dependent on glutamine metabolism, both as a fuel for TCA cycle and precursor of gluthatione necessary for ROS scavenging, and glutaminolysis inhibition rescues resistance to FLT3 inhibitors (27). Moreover FLT3-ITD AML models demonstrate increased levels of aerobic glycolysis and similar effects in enhancement of the activity of FLT3 inhibitors can be achieved by glycolysis inhibition (69). FLT3-inhibition induces a marked upregulation of cellular oxidative stress which is circumvented through the activity of glucose 6-phosphate dehydrogenase. The inhibition of this enzyme and the use of compounds that increase mitochondrial ROS, like phenformin, greatly increased the sensitivity to FLT3-inhibition (70). In addition, it was recently described that patients with FLT3-ITD mutation present with dysregulated serine metabolism and inhibition of *de novo* serine synthesis, most likely through decrease in purine synthesis for which serine is a physiological precursor, decreases proliferation of FLT3-ITD mutated AML cells and increases their sensitivity to cytarabine (26).

Many other molecular markers appear to be associated with specific dysregulation in metabolism. Patients with t(8;21) translocation often present with mutations in transcription factor ZBTB7A (71), which has been described as transcriptional repressor of several critical glycolytic genes (72). AML cells with mutated *EVI1*, an oncogene associated with particularly poor clinical outcome, depend on mitochondrial creatine kinase CKMT1. Inhibition of CKMT1 induced a blockade in arginine–creatine pathway and impaired mitochondrial respiration and ATP production. Interestingly, the gene sets that were most enriched in AML cells overexpressing *EVI1* were purine and pyrimidine synthesis pathways (73). Specific alteration in polyamine, purine and pyrimidine metabolism have also been described in patients with *NPM1*-mutations, a marker associated with favorable prognosis (74), again pointing out to a more widespread and under-investigated role of nucleotide synthesis in AML. A preliminary report in a mouse model demonstrated that induction of *ASXL1* mutation in hematopoietic stem and progenitor cells induced hematopoietic dysfunction followed by an increase in mitochondrial activity and ROS production (75), which might indicate for a potential role of metabolic dysregulation in pathogenesis of *ASXL1*-mutated AML. It is also worth noting that cancer cells with mutations in *SF3B1*, a gene which is frequently mutated in myelodysplastic syndrome and chronic lymphocytic leukemia, demonstrated dysregulated serine synthesis pathway *via* downregulation of enzyme phosphoglycerate dehydrogenase (PHGDH) and increased dependency to extracellular serine (76), which is interesting because of the role serine plays in one carbon metabolism which contributes to folate metabolism, synthesis of nucleotides and several amino acids, as well as methylation reactions and redox balance (77). Finally, one of potentially most intriguing patient subgroups for metabolic targeting is the one with *TP53* mutations. *TP53* mutations are commonly observed in solid tumors, but are present in only 5–10% of AML patients and, in spite of some recent progress in targeted therapy (78), they still represent a very unfavorable prognostic marker without an adequate treatment option (79). *TP53* has been shown to regulate a multitude of metabolic pathways in solid tumors, including glucose metabolism, oxidative phosphorylation, lipid metabolism, nucleotide metabolism etc. (80), but very little is known on its potential role in metabolic regulation in AML and further work is needed to elucidate if metabolic targeting could result in novel therapeutic strategies for AML patients harboring *TP53* mutations.

**Figure 1:**
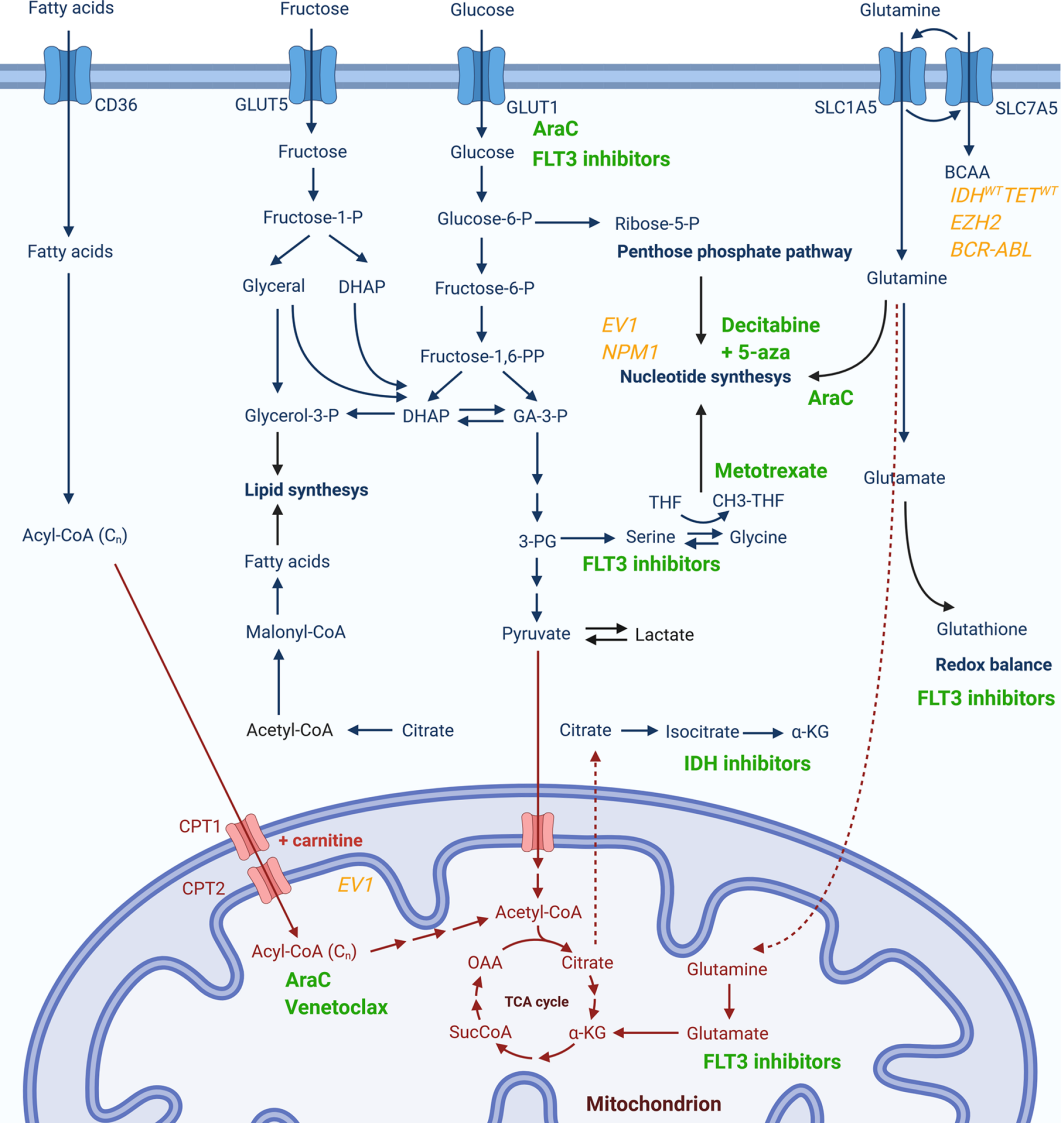
Metabolic pathways potentially associated with resistance to currently used pharmacological agents or specific genetic signatures. Chemotherapeutics or targeted therapy drugs currently in use for AML (in green) are presented next to the cytosolic (in blue) or mitochondrial (in red) metabolic pathway whose activity has been reported to be modulated upon treatment with respective compound or which has been associated with drug resistance. Genetic markers associated to changes in certain metabolic pathways are given in yellow.

The group of patients that could most likely benefit from metabolic modulations are patients resistant to available therapies. As already mentioned, resistance to both cytarabine (47)⁠ and venetoclax (44, 45)⁠ are mediated by increased OxPhos and metabolic switch to FAO, while resistance to FLT3 inhibitors is mediated through alterations in metabolism of glutamine (27)⁠ and serine (26), as well as glycolysis (69). During intensive chemotherapy, AML cells appear to pass through a form of metabolic bottleneck producing resistant cells with a distinctive metabolic signature consisting of a switch in glutamine utilization primarily for nucleotide synthesis and gluthatione production and a modification of pyrimidine synthesis pathway to a higher dependency from extracellular aspartate provided by the bone marrow stroma (81). A targeted metabolic disruption could, thus, serve as a manner to prevent this metabolic plasticity of leukemic cells, overcome the survival advantage it provides, and reduce the establishment of resistance and consequent relapses. Metabolic pathways that could be relevant for resistance to pharmacologic agents in use, together with molecular signatures potentially associated with certain metabolic dysregulations, are summarized in **Figure 1**.

No compounds directly targeting an unmutated metabolic pathways has been approved for the treatment of AML, but there are several currently in clinical trials. Phase I clinical trials are underway for the glutaminase inhibitor CB-839 (NCT02071927) and oxidative phosphorylation inhibitor IACS-010759 (NCT02882321) in patients with relapsed or refractory AML (82), as well as safety and tolerability of adding HMG-CoA inhibitor pitavastatin to venetoclax in subjects with newly diagnosed AML (NCT04512105). After promising preclinical data that showed reduction in AML burden upon depletion of extracellular arginine levels using pegylated arginine deiminase (83), a phase II study in 43 relapsed/refractory/poor-risk patients demonstrated only a modest response (84) and a randomized trial of pegylated‐recombinant arginase I BCT‐100 in elderly AML patients is currently ongoing in the United Kingdom (unpublished correspondence).

Another intriguing therapeutic strategy for targeting metabolic pathways in AML could be through pharmacological modulation of their upstream regulatory signaling pathways, many of which have inhibitors already in clinical use (85). As one example, the mTOR pathway, which is a long recognized regulator of glucose, lipid and amino acid metabolism, has been recently re-evaluated for AML treatment in combination with standard chemotherapy when given in specific timed manner (86). Due to the breadth of the field of signaling in metabolic regulation, we refer the readers to other dedicated reviews on the topic (85, 87, 88).

Finally, the potential of dietary modifications and nutritional supplementation in unmasking the vulnerabilities of AML cells is recently being increasingly investigated. High fructose diet is known to contribute to cancer development. However it can also unmask novel vulnerabilities as AML cells upregulate *de novo* serine synthesis pathway in high fructose conditions, and the inhibition of one of the components of this pathway, phosphoglycerate dehydrogenase, can block the reliance on fructose utilization (89). It is yet to be fully determined whether the reduction of fructose in the diet of AML patients could also prove to be of clinical efficacy (41), or it is a wider epidemiological target in the attempt to reduce the general burden of cancer (90). Additionally, specific dietary intervention can also be devised to modulate metabolic pathways which can enhance the therapeutic efficacy of standard therapies. As an example, preclinical evidence has shown that supplementation with methyl-tetrahydrofolate (91) or histamine (92) show beneficial anti-leukemic effects by modulation of folate pathway which enhances the the efficacy of MYC-targeted therapies and methotrexate, respectively.

## Conclusions

Precision medicine has become an increasingly desirable goal in oncology. Using available knowledge banks of matched genomic-clinical data has already been shown to facilitate personally tailored therapeutic decisions (93). Moreover, the recent development of targeted therapies towards specific genetic subgroup will further increase the efficacy and reduce the toxicity of such personalized therapeutic approaches. Unique metabolic rewiring in several AML subtypes/cell populations and its role as therapeutic targets or in therapy resistance has increasingly come into focus. It is therefore possible to envision a near future in which we will be able to incorporate metabolic analysis in our knowledge banks to further refine our ability to devise personally tailored therapies. Moreover, the development of “targeted” metabolic inhibitors shown to be effective, specifically in certain AML subtypes, will also increase our therapeutic options for unique patient groups. Finally, our improved understanding of metabolic changes in AML can also be leveraged by using tailored dietary interventions likely to improve the efficacy of specific drugs thus providing a truly holistic approach to patient management.

## Author Contributions

VD and PG carried out primary literature search and wrote the manuscript. All authors contributed to the article and approved the submitted version.

## Funding

Both authors are currently funded through a CRUK Advanced Clinician Scientist Fellowship reference C57799/A27964.

## Conflict of Interest

The authors declare that the research was conducted in the absence of any commercial or financial relationships that could be construed as a potential conflict of interest.

## Abbreviations

5-aza, 5-azacitidine; AraC, cytarabine; IDH, isocitrate dehydrogenase; FLT3, Fms Related Receptor Tyrosine Kinase 3; 3-PG, 3-phosphoglycerate; α-KG, α-ketoglutarate; BCAA, branched-chain amino acids; CH3-THF, methyltetrahydrofolate; CoA, coenzyme A; CPT, carnitine palmitoyltransferase; DHAP, dihydroxyacetone phosphate; GA-3-P, glyceraldehyde 3-phosphate; GLUT; glucose transporter; OAA, oxaloacetate; P, phosphate; SLC, solute carrier transporter; SucCoA, succinyl-coenzyme A; THF, tetrahydrofolate.
